# Duration judgments are mediated by the similarity with the temporal context

**DOI:** 10.1038/s41598-022-27168-w

**Published:** 2022-12-30

**Authors:** Jaume Boned, Joan López-Moliner

**Affiliations:** Vision and Control of Action (VISCA) Group, Department of Cognition, Development and Psychology of Education, Institut de Neurociències, Universitat de Barcelona, Barcelona, Catalonia Spain

**Keywords:** Human behaviour, Computational neuroscience

## Abstract

When we try to assess the duration of an event, we are often affected by external information. Studies on multiple timing have found that simultaneous timing information can produce an averaging or central tendency effect, where the perceived duration of the elements tends to be biased towards a general average. We wanted to assess how this effect induced by simultaneous distractors could depend on the temporal similarity between stimuli. We used a duration judgment task in which participants (n = 22) had to compare the duration of two identical targets (1 s) accompanied by simultaneous distractors of different durations (0.3, 0.7, 1.5 or 3 s). We found a central tendency effect, where duration judgments of the target were systematically biased towards the duration of the distractors that accompanied them. We put forward a model based on the concept of duration-channels that can explain the central tendency effect with only one estimated parameter. This parameter modulates the rate of decay of this effect as distractors duration become more different than the duration of the target.

## Introduction

Perceiving the duration of the events around us is a key component of how we experience the world. Research in time perception has focused on many factors that can make an event seem to last longer or shorter^1–4^. However, distortions of duration can be caused not only by the intrinsic properties of such events but also by the presence of external but concurrent stimulation. In real-life situations, events are not quite isolated in the temporal dimension. When we attend to some event that is taking place during a specific time interval, other events can often occur simultaneously in the same scene. The durations of these concurrent events are potential sources of perceptual noise that can affect how we experience the duration of the main event we are trying to focus on^1,5,6^. This concurrent information, referred to as the temporal context, includes all that temporal information in the same environment as the event to be perceived. For example, the duration of a red traffic light for which we are waiting might not be perceived as lasting the same when isolated as when there are many other traffic lights constantly changing at their own pace for different lanes.

Therefore, this temporal context can refer to simultaneous information about time such as duration, frequency, or synchrony to other surrounding elements and to previously, or even posteriorly, experienced information related to that same event.

To begin with, studies focusing on the contextual effects of preceding information proved that our estimations are not independent of previously presented information. For example, Hallez et al.^7^ studied the impact of temporal context on a temporal reproduction task. Under the Bayesian theory of perceptual inference of time, they expected that reproductions of duration could be affected by previous experience (prior) with different durations. Specifically, they found that reproductions of the same duration tended to be overestimated when presented in an array of stimuli with longer durations and underestimated when such array was of shorter durations. They suggested that as compensation when facing uncertainty, time reproductions relied more on prior information, which induced a central tendency effect in which estimations were averaged towards the values of its temporal context^6,7^.

The same result is usually found in “carryover effect” studies on duration judgments^8^, but more importantly, these effects include the influence of both previously presented stimuli and previous decisions. Interestingly, the direction of this effect is shifted between decisional and perceptual carryover. For example, central tendency or assimilative effects are usually found from previous duration judgments, whilst previously presented durations can produce a repulsion effect, where current durations are perceived as more different in magnitude than previously presented stimuli^8,9^.

Even when previous information is not relevant to the task, it can also affect posterior duration judgments. Burr et al.^10^ examined how temporal context could affect duration judgments by presenting contiguous distractors immediately prior to a target. They found that duration judgments of the target were systematically biased following an assimilative tendency; when distractors of short duration accompanied the target stimuli, they were perceived as shorter and vice versa. Moreover, if signals of the same modality delimited each interval, the effect would occur even when distractors were presented immediately after the target, instead of previous to it. It was also reproduced in both visual, auditory and tactile modalities. Additionally, they found that when distractors were far greater than the target, the interaction between their durations would be substantially reduced. This finding could be addressed from the framework of a channel-based model described as a system of duration-selective channels (neuronal populations) that are differentially activated by specific durations^11,12^. More specifically, these channels respond preferentially to certain intervals based on the time between the onset and offset of an event^13^. This type of tuning to specific ranges of stimulation has already been observed for other kinds of information, such as visual orientation or auditory pitch^11,14^. There is evidence from animal studies of neurons that are tuned to a preferred duration^14^. Using fMRI in humans, it has been shown that adaptation to a time interval reduces neural activity in specific areas when identical durations were repeated, which provides evidence of duration-tuned neural structures in humans^15^.

From this framework, Heron et al.^11^ used adaptation techniques to show how repeated presentations of a stimulus can affect the estimated duration of following stimuli of the same or similar duration. They also found that when adapted and test durations were different enough, the effects would fade, which could be parallel to the findings of Burr et al.^10^. They argued that the duration-selective channels could be the ones to be adapted. Although contrary to the assimilative effects mentioned earlier, adaptation after-effects of duration often result in a repulsive effect, where perceived times are shifted away from the adapted durations.

So far, we have only described effects from serial timing information however, temporal context also includes information about the duration of simultaneous events. From the "Multiple Timing" framework, first described by Brown and West as the process involving the capacity to attend and extract duration information from multiple sources^16^, studies have explored how overlapping intervals could influence duration estimations.

For example, Kawahara and Yotsumoto^17^ found that when stimuli were presented along with distractors, duration reproductions tended to be overestimated when such distractors lasted longer than the interval to be reproduced and underestimated when distractors lasted less than the target interval. This finding is similar to those reported above about the carryover effect and Bayesian studies; an averaging effect that takes place between the duration of a target and the duration of the rest of the elements that constitute the temporal context^18–20^. It has been argued that the increase in perceptual noise might elicit a mandatory and automatic averaging between the durations of the different simultaneous stimuli of a sequence, regardless of their relevance or attentional demand^18^.

Finally, when comparing the effect of sequential and simultaneous presentation of durations in the temporal context, they do not differ substantially^17,18,20^. At most, when stimuli are overlapped, performance is in some cases worsened, probably due to an increase in the immediate perceptual noise^20,21^. Note that this interaction between pieces of information that we describe as perceptual noise represents, in this case, a nuisance for discriminating the exact properties of a specific element, although it could prove useful when trying to extract some general statistics from an array^22^.

In the present study, we wanted to test whether duration judgments of elements surrounded by simultaneous irrelevant distractors could be influenced by this immediate temporal context and discern what kind of effect would take place by this type of presentation. We also wanted to test whether the effect of temporal context depends on the degree of similarity between stimuli and see whether the duration-channels framework provides a good explanation.

To this aim, we designed a comparison judgment task in which the durations of two target stimuli are compared while other distractor stimuli with different durations are presented simultaneously. We generated two sequences, each composed of a target and a set of distractors. By using the same duration for both targets, we could expect that any systematic difference in judged duration between them should be due to the influence of the presented distractors overlapped in time with them, that is to say, the effect from the temporal context. This rationale is analogous to the well-known size-contrast perceptual illusions that produce different perceptions of the same stimuli by manipulating the surrounding stimuli. More specifically, our task is the temporal version of the Ebbinghaus-Titchener illusion^23,24^, where the size of surrounding stimuli affects the size judgment of the central target. We used this same approach in the temporal domain by presenting targets of equal duration but overlapped with distractors of longer or shorter duration and asking participants which of the two targets seemed to last longer. This approach can be regarded as deceptive since in one condition the two targets lasted the same. However, we relied on the distractors affecting the perceived duration of the central target, making them to be perceived differently. After questioning the participants at the end of the experimental sessions, we can ascertain that our manipulation did not seem to make them feel deceived.

If distractors induce an effect in the same way as those reported in Bayesian studies, carryover effects and the findings of the studies on Multiple Timing, targets should seem to last longer when accompanied by distractors of longer duration and vice versa. Finally, considering the duration-channels theory, we expect such effects to fade as durations become more and more different to the duration of targets.

Therefore, we outline a model that assumes a leaking factor that determines the rate at which temporal context effects would fade (or not) as the elements become more and more different. Estimating this leaking factor could allow for a better understanding of how duration representations of multiple simultaneous elements are combined during a judgment task and how their similarity in duration might modulate this effect.

### The duration-channel leaking model

In order to address how the duration of distractors could influence duration judgments of a target with which they are overlapped in time, we elaborate a computational model based on the duration-channels theory^11^.

The first assumption is already proposed in the duration-channels theory^11^ and defines that there exist neural paths (channels) which respond selectively to specific time intervals. Based on distractors and Multiple Timing research, we also expect that unattended stimuli can activate these channels and that concurrent stimuli with different durations should activate channels sensitive to each duration.

Since distractors are known to systematically bias duration estimations, we propose that the information associated with this parallel activation of channels from distractors and targets might be integrated into the estimated target duration. Considering previous findings on simultaneous timing, we expect this influence to reveal an averaging or central tendency effect. Therefore, the perceived duration of a specific element should be shifted towards the general average of the different durations presented at the same time.

Additionally, this model considers the possibility that the magnitude of this shift depends on the similarity in duration between stimuli, that is, how close the activated channels are with one another.

**Figure 1:**
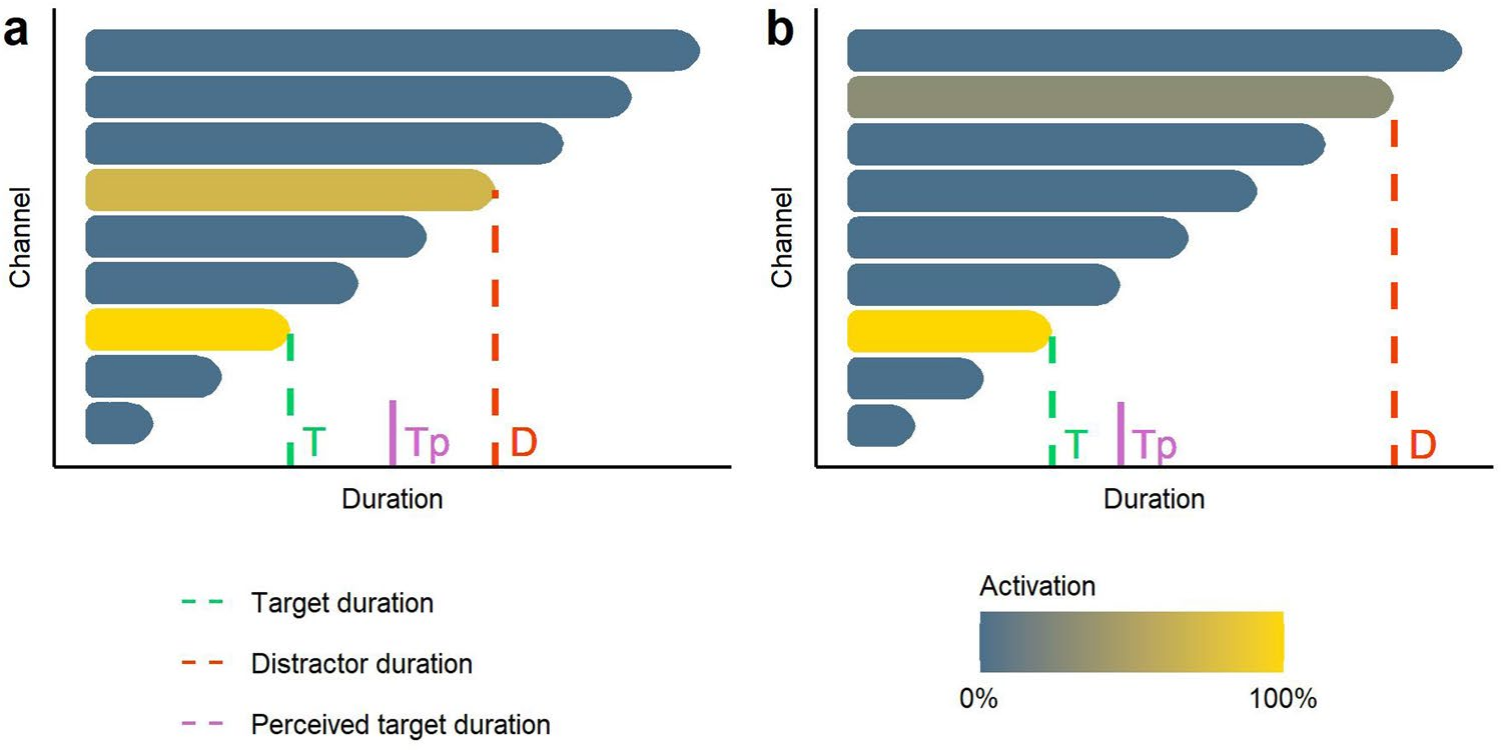
Illustration of the duration-channels activation process. When a target (T) is presented with a distractor (D), both activate the channels associated with their respective durations. The perceived duration of the target (Tp) is shifted towards the duration of the distractor. This information leaking has a stronger effect when distractor and target durations are more similar (**a**) and decays when they are more different (**b**).

In summary, we expect distractors to activate the channels associated with their durations. This activation would be integrated into the perceptual processing of a target as a form of information leaking. The strength of this leaking would be smaller when the target and distractor durations are more different. See Fig. 1 for a visual representation of this leaking process assuming the integration of duration-channels activation as a weighted average.

The function that computes this integration consists of a weighted average that combines both target and distractor durations. The influence of distractors is modulated by a weighting function that depends on the difference between target and distractors durations. The function of this weighted average is defined as:

1 $$\widehat{t} = \frac{{\left( {t + d \cdot w} \right)}}{{\left( {1 + w} \right)}}$$

It averages the target (t) and distractors (d) durations with a weight (w) that will always be maximum (w = 1) for the target since it is the relevant duration of the perceptual task. The weight of the distractors will range between 0 and 1. The greater the weight of distractors, the more influence they will have on the perceived duration of the target.

**Figure 2:**
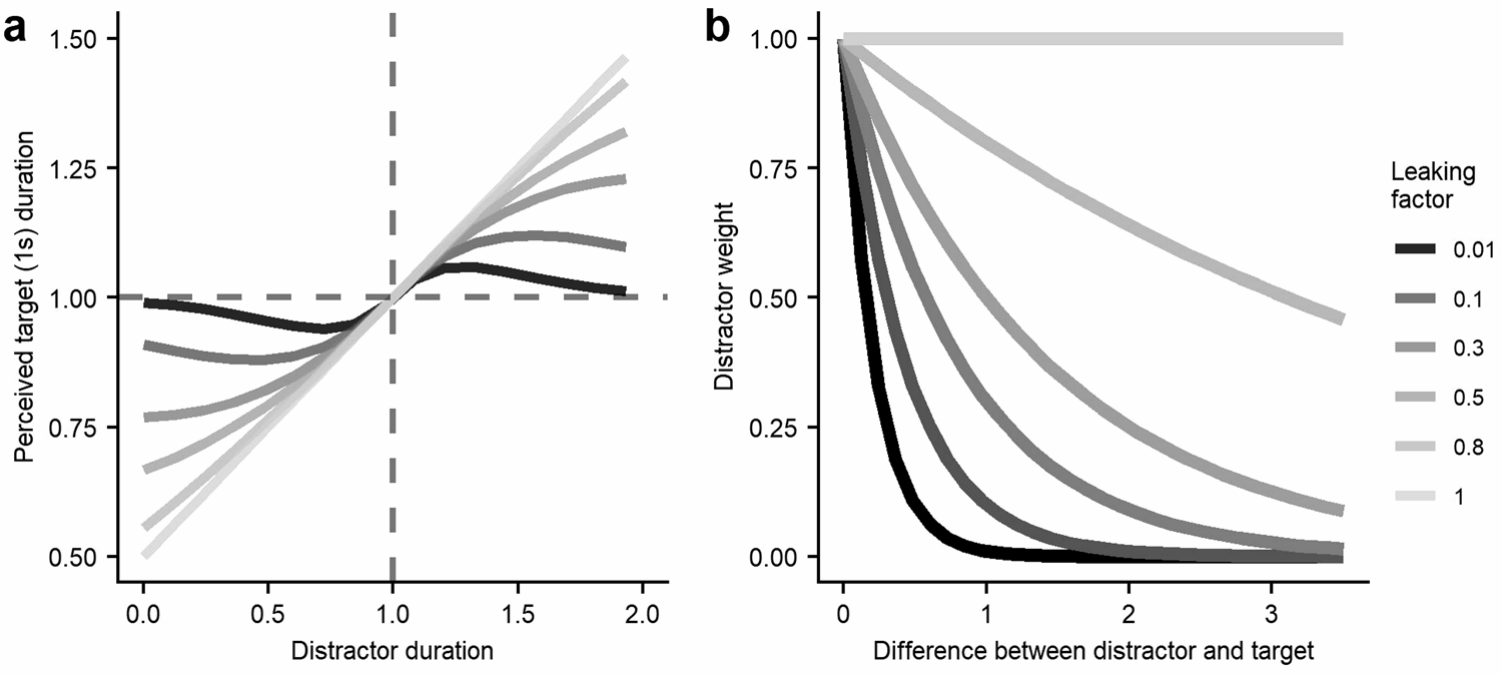
(**a**) Perceived duration of a 1 s target as a function of the duration of simultaneous distractors. (**b**) Weight of the distractors as a function of their difference with the target duration. Each line represents a different value of the leaking factor k.

The weight of the distractors is calculated from the following function, named leaking function:

2 $$w = k^{{\left| {d - t} \right|}} ;{ }0 \le k \le 1$$

where the weight (*w*) will result in positive values from 0 to 1. Durations of distractor and target are represented by *d* and *t,* respectively, and the absolute difference between them is the exponent of the function. The leaking factor (*k*), with values between 0 and 1, determines the rate at which the weight would decrease as distractors and target become more different. With a *k* of 1, distractors will always have the same weight (w = 1) regardless of their difference with the target. When *k* takes values smaller than 1, the weight will decay as the difference becomes greater, meaning that distractors very different from the target will have a smaller weight in the averaging. Specifically, the closer *k* is to 0, the faster the weight will decay as the difference increases (see Fig. 2), getting substantial weight only for those distractors sufficiently similar to the target. In all cases of *k*, when the exponent is 0, meaning no difference between target and distractors, the weight will be maximum, but it will make no difference in the resulting perceived duration of the target since the average between stimuli will be the same as the duration of the target alone in the first place.

Figure 2 shows how the perceived duration of a target (duration = 1 s) varies with distractors of smaller or greater durations (y-axis) with different levels of the leaking factor. The leaking factor is a free parameter, and its value can be estimated with a fitting procedure for each.

Since Weber's Law is one of the most well-established findings in time perception, we must address how the proposed model relates to Weber’s law. The model (Eq. 1) implies a linear relation between physical time and perceived time ($\widehat{t}=t$) in the absence of distractors (*d* = 0 and *w* = 0). This could seem to be at odds with Weber’s Law which states that usually, but not always, we have a perceptual scale in which the perceived magnitude is a logarithmic function of the physical stimulus, as stated by Fechner’s law (e.g. $\widehat{t} = {\text{k}}^{\prime } {\text{log}}t$ + C). In our case, although our model predicts a linear relation instead, Weber’s Law could still hold under the assumption that the internal noise increases with the duration magnitude of *t*, that is the noise would be signal-dependent or multiplicative^25^. In this case, the predicted JND pattern ($\Delta t$ that is noticeable) would also increase proportionally with physical time.

Equation (1) also allows us to predict the perceived difference between a perceived duration (after combining a physical duration with distractors) and the same physical duration without distractors. In Fig. 5 of Supplementary material we show how this perceptual increment varies as a function of distractor duration for different target durations and leaking factors.

## Methods

### Participants

The sample consisted of twenty-two participants from the University of Barcelona. All of them gave written informed consent to participate in the experiment and were initially naïve to the purpose of the experiment. Fifteen participants were female, and seven were male (mean age = 25.23, SD = 2.89). All of them had normal or corrected vision. Due to the COVID-19 pandemic lockdown, eight of the participants performed the experiment online using the Pavlovia platform, an online hosting service that allows for running Psychopy-based experiments. The study is part of a research program that has been approved by the ethical committee of the University of Barcelona (IRB00003099) according to the principles stated in the Declaration of Helsinki.

### Apparatus and stimuli

The task was conducted in laboratory conditions using Psychopy software^26^ in a Mac Pro. Stimuli were presented on a 24.5 inches ASUS ROG Swift PG258Q monitor using a resolution of 1920 × 1080 pixels and at a 240 Hz refresh rate. Participants used a chinrest at 57 cm of the screen.

**Figure 3:**
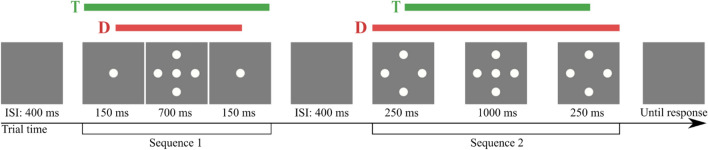
Example of experimental trial with 700 ms distractors (sequence 1) paired with 1500 ms distractors (sequence 2), each centred in time with 1000 ms targets.

The visual stimuli were white blobs (maximum luminance of 226.2 cd/m2) with a raised cosine mask of a full diameter of 4.5 deg and a central plateau of 4 deg against a grey background (mean luminance of 55.38 cd/m2) (see Fig. 3). They could appear either one alone in the centre of the screen or along with four more on the left, right, top and bottom of the centre, respectively, with 9 deg of eccentricity.

### Procedure

#### Duration judgment task

Participants were asked to observe two sequences presented serially and compare the duration of specific elements from each sequence (see Fig. 3).

After observing the two sequences, participants had to respond by pressing the left arrow key of the keyboard if they perceived the target stimuli from the first sequence as lasting longer or the right arrow key if they perceived the target stimuli from the second sequence as lasting longer. Each sequence was preceded by a 400 ms blank interval, as well as between the last sequence and the response phase.

Three different conditions were used within participants, which determined which elements were the target stimuli to be compared.

In the **Distractors** condition, the sequences consisted of a central target and four surrounding blobs (distractors). Participants were asked to compare the duration of the central targets exclusively and ignore surrounding ones. The duration of the central target was always 1000 ms for both sequences. In each sequence, the duration of all four distractors was homogeneous between targets of the same sequence, either 300 ms, 700 ms, 1500 ms or 3000 ms, but always different between the two sequences of a pair. Considering that target and distractors would always have different durations, the onsets and offsets were adjusted so that durations were centred. See Fig. 3 for an example.

In the **Ensemble** condition, we used the same stimuli as in the **Distractors** condition, with the only difference that the instructions were to compare the duration of the whole array of stimuli from each sequence, considering the target to start with the appearance of the first element and end with the disappearance of the last one, regardless of them being the central or surrounding elements. Hence, all stimuli were considered part of the target in this condition.

Finally, in the **Control** condition, each sequence consisted of only one central blob (target). The target duration in each sequence could be 300 ms, 700 ms and 1500 ms in any combination but always different between sequences of a pair.

In each condition, all possible combinations of sequences were repeated up to 30 times per participant and divided into five blocks that included six repetitions of each combination in a randomised order. After each of the blocks, participants could take a short self-timed break. Each participant performed all three conditions of the task in a counterbalanced order.

### Predictions

We will use the **Control** and **Ensemble** conditions to check whether participants are able to discriminate durations well enough in this task. We will then analyse the psychometric functions of each participant. Also, by comparing the performance of both conditions, we might be able to look for differences in duration discrimination when the time intervals consist of either one or multiple elements.

We will then check whether the distractors affected the judged durations in the **Distractors** condition. We expect the distractors to induce a central tendency effect. By fitting a psychometric curve to the responses of this condition as a function of the difference between distractors, we will derive the slope signed value. The sign can be used as an indicator of either repulsion (negative), or central tendency (positive).

If a central tendency effect is found, we will proceed with the Channel Leaking model to obtain each participant's leaking factor and check how well the model describes the response pattern.

Additionally, we want to address whether the leaking factor could be related to the discrimination capacity when time intervals include multiple elements. In our case, the leaking factor measures how participants are affected by distractor durations, which could be considered a source of noise. We predict that a smaller leaking factor, which would suggest a better inhibition of the perceptual noise introduced by distractors, should be related to better performance when measured with discrimination thresholds. Hence, they should be inversely correlated.

## Results

### Duration judgements performance

First, we checked whether participants were able to discriminate between the different durations that we will be using for the distractor stimuli.

**Figure 4:**
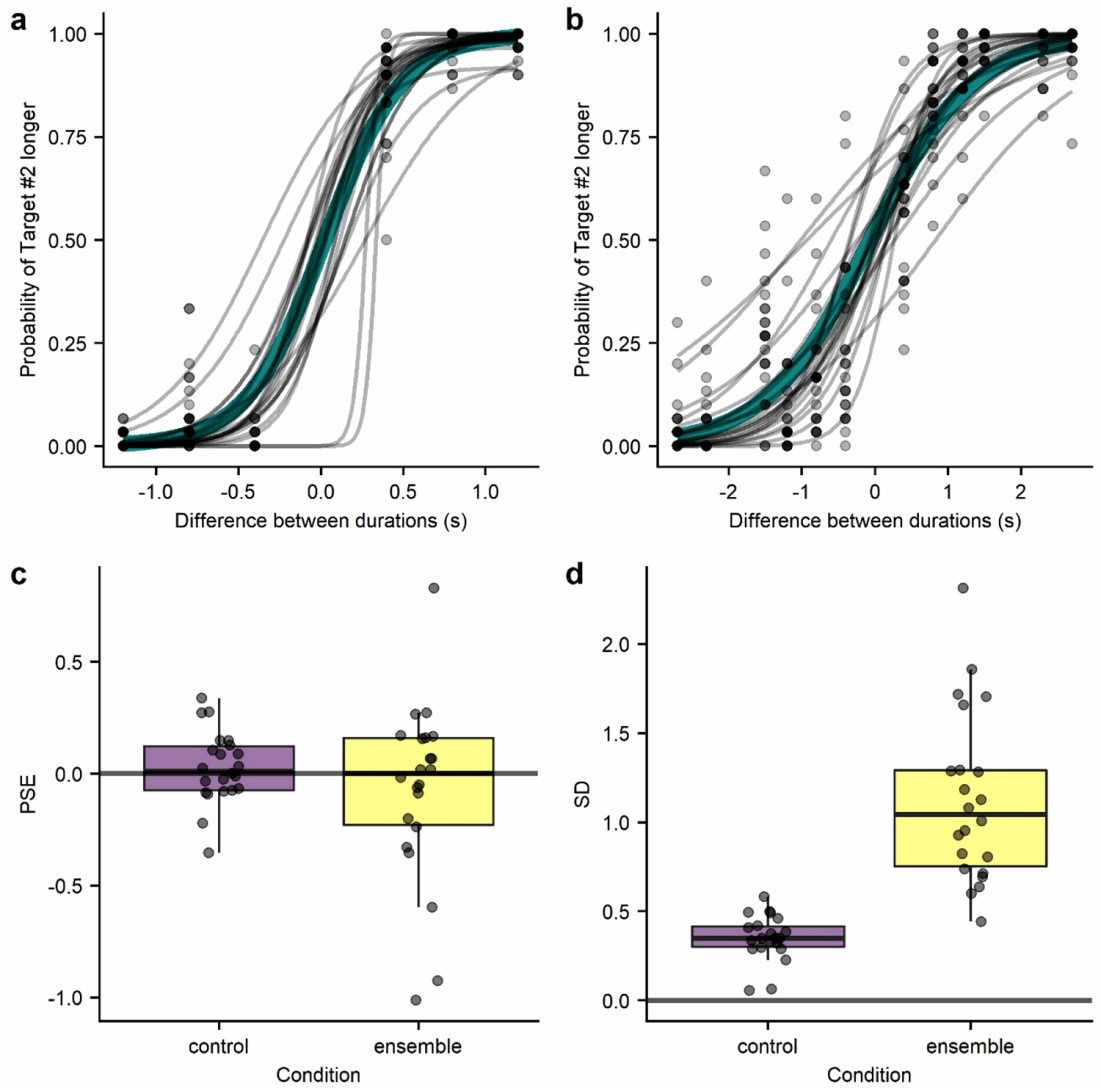
Psychometric curves of every participant in the control condition (**a**) and ensemble condition (**b**). Each grey line represents a different observer, and green thicker lines show the fit to the aggregated data from all participants. Comparison between the estimated Point of Subjective Equality (PSE) (**c**) and Standard Deviation (SD) (**d**) of the psychometric curves from each participant in the control and ensemble conditions.

We fitted psychometric curves using the logistic function to the Ensemble and Control conditions data. Parameter estimates of the curves were obtained with MLE using the Quickpsy package^27^ for R software^28^ and the 95% confidence interval by using bootstrap^29^. Data were fitted individually for each participant, including the point of subjective equality (PSE) and the slope of the curve as free parameters. Since the value of the PSE and mainly the slope, can be affected by lapse rates^30,31^, we included the lapse rates parameter and kept the PSE and slopes values that corresponded with the best model (with versus without lapses) based on the Akaike information criterion (AIC). Then, we obtained a measure of precision for duration discrimination. We derived the standard deviation of the psychometric function from its slope. It represents how good observers are in differentiating between durations, with smaller values related to better sensibility. Both models were fitted with the proportion of “Second interval lasted longer” responses as a function of the difference between durations in seconds (second interval – first interval). See Fig. 4 for the psychometric curves (Fig. 4a, b) and their parameters' estimates (Fig. 4c, d).

Participants were able to discriminate well between the presented durations in both the Control (mean percentage of correct responses = 95%) and Ensemble conditions (mean percentage of correct responses = 86%).

Additionally, the standard deviations of the psychometric curves were greater (t(21) = − 8.657, *p* < 0.001) in the Ensemble condition (mean = 1.13,SD = 0.477) than the Control (mean = 0.35,SD = 0.126) condition. This finding suggests that participants needed greater differences between durations for correct discrimination in the Ensemble condition, where the intervals were composed of multiple elements. Then, this decline in discrimination could be associated with an increase in perceptual noise.

The PSE of the curves informs us of any general bias in the duration judgements. Globally, there is no clear pattern of bias between participants (Fig. 4c). Still, there is some variability, with participants judging either the first or the second interval as longer or showing no preference.

### Effect of distractors

We used the same method to fit the psychometric function of each participant from the Distractors condition. Here, we wanted to assess whether distractors duration could affect the judged duration of the targets. Responses were fitted to the psychometric curve that describes how the probability of responding “The target within the second sequence lasted longer” may vary as a function of the difference between distractors (distractor duration from the second sequence—distractor duration from the first sequence). These values are the same differences that we compared at the Ensemble condition. Note that the aim of fitting psychometric curves in this condition is to reveal any effect of the distractors on the judgement of the one-second targets. However, since we have no physical variation of the relevant stimulus (i.e. target duration), we should not interpret the parameters of the fit in the same way as in the Control or Ensemble condition.

The slope of the fitted curves will then be used to assess the type of effect caused by the distractors. A positive slope would mean that targets surrounded by distractors of longer duration are more likely to be judged as lasting longer; we will refer to this effect as a central tendency. On the contrary, if the slope is negative, it would mean that targets surrounded by shorter duration distractors are more likely to be judged as lasting longer; we will refer to this effect as repulsion tendency. The other possibility is that participants' probability of judging a target as lasting longer is not affected by the duration of distractors, which would result in a null slope (not different from 0).

**Figure 5:**
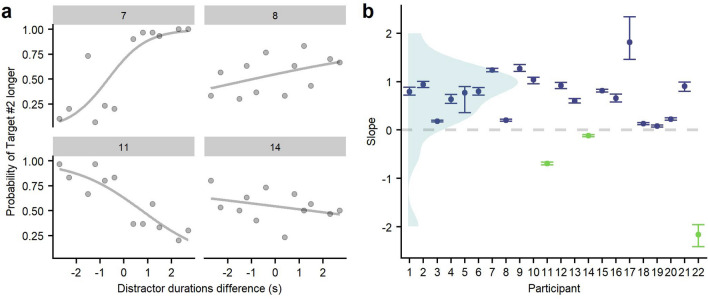
(**a**) Psychometric functions from a sample of four participants (7, 8, 11 and 14) with high (left column) and low (right column) slopes as well as positive (upper row) and negative (lower row) values. (**b**) Distribution and slope values of the psychometric curve of each participant in the Distractors condition. Points represent the slope estimate and brackets represent 95% confidence intervals.

For all participants, the 95%-CI of the slope estimate did not include 0. Therefore, their judgments of the central target did vary as a function of the duration of distractors. In 19 participants out of 22 we obtained positive slope values, indicating a central tendency, where target durations were judged as more similar to the duration of the distractors in their sequence. Alternatively, three participants showed negative slope values, indicating a repulsion tendency in which the judged durations of targets were shifted away from the duration of the distractors that accompanied them (See Fig. 5, or S1 in Supplementary Material for data from all participants).

Since most participants exhibited a central tendency, and it is part of the assumptions of the Channel Leaking model, those participants with repulsion tendencies will not be included in the estimation of the leaking factor based on Eqs. (1) and (2).

### Duration-channel Leaking model

With the data from those participants that manifested a central tendency, we estimated the leaking factor (k) (Eq. 3) by using MLE that predicts the probability of response judgement for each combination of distractor durations within participants. The optimization procedure maximized the log-likelihood for k across the different combination of distractors duration *S*:

3 $$LL\left( k \right) = \mathop \sum \limits_{i = 1}^{S} \left( {\begin{array}{*{20}c} {n_{i} } \\ {m_{i} } \\ \end{array} } \right) + m_{i} \cdot{\text{log}}(\varphi_{k} ) + \left( {n_{i} - m_{i} } \right)\cdot{\text{log}}\left( {1 - \varphi_{k} } \right)$$

$n_{i}$ denotes the number of presentations of each combination of distractors and $m_{i}$ the number of times a participant judged the duration of the second target as the longest. $\varphi$ denotes the standard Gaussian distribution (mean of 0 and SD = 1) which takes as an argument the difference of perceived durations between the two targets:

4 $$\varphi_{k} = {\mathcal{N}}\left( {\hat{t}_{2} - \hat{t}_{1} } \right)$$

The leaking factor *k* is actually embedded in Eq. (4), since each perceived target duration is defined as previously introduced in Eqs. (5) and (6):

5 $$\widehat{t} = \frac{{\left( {t + d \cdot w} \right)}}{{\left( {1 + w} \right)}}$$

6 $$w = k^{{\left| {d - t} \right|}} ;{ }0 \le k \le 1$$

**Figure 6:**
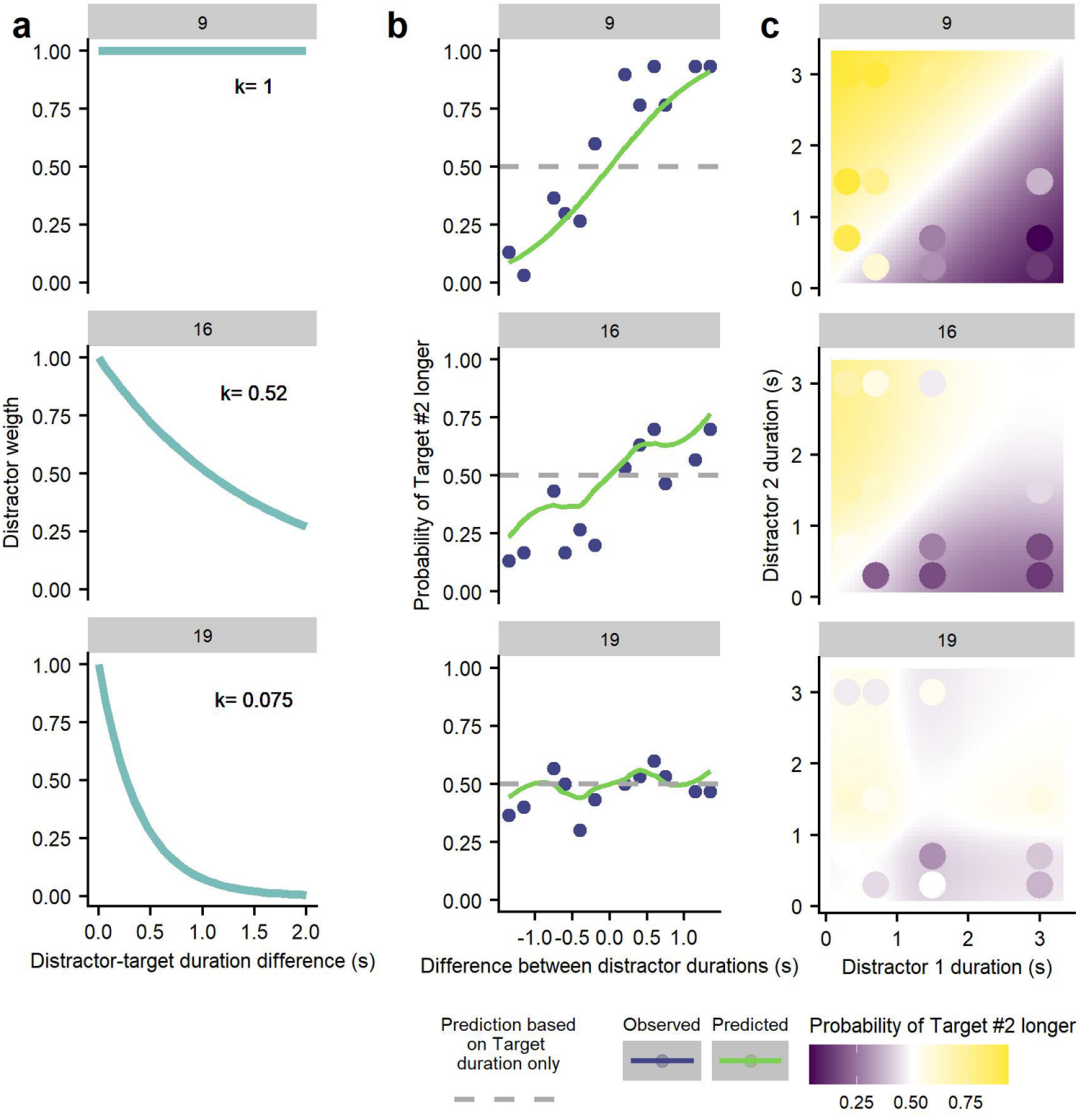
Sample of three participants (9, 16 and 19) with high, medium and low values of leaking factor. (**a**) The weight function of the leaking factor predicts how much weight the distractors will have depending on their similarity with the target. (**b**) Fit between predicted and observed response probabilities as a function of the difference between distractors. (**c**) Comparison between observed (coloured circles) and predicted (background colour) probabilities of response for any combination of distractors.

We then obtained the *k* parameter for each participant, which allows us to describe the weight of distractors as a function of their difference in duration towards the target. We found a wide range of *k* values between participants, from 0.006 to 0.999 (mean = 0.46, SD = 0.312). Then, we compared the predicted response probabilities with the observed data to measure the goodness of fit (See Fig. 6). Figure 6a shows the predicted distractors weight (w) functions for three participants with high, medium and low values of estimated k values. These curves show how the weight of the distractor decreases (y-axis) as the difference between target duration and distractor duration increases (x-axis) and how this rate of decrease varies with the leaking factor. Figure 6b shows the observed duration judgements (dots) along with the predicted (solid line) probabilities of judging the target of the second sequence as longer (y-axis) as a function of the difference in durations between distractors of each sequence (x-axis) obtained from Eq. (4). As represented by the grey dashed line, if distractor durations are successfully disregarded or are not even presented, the predicted probability of judging target #2 as lasting longer would be 0.5. In Fig. 6c, we show how the predicted probability of judging the second target as being longer (represented in a colour scale) can vary for any combination of distractor durations (x and y-axis). One can see how, for the same combination of distractors duration, the leaking factor (*k*) biases perceived target duration differently depending on the *k* value. The heat maps denote the predicted response probabilities, and the circles denote the observed probabilities. The more similar the colours between circles and background, the better the fit. See Fig. S2, S3 and S4 in the Supplementary Material for the same type of plots with data from all participants.

We performed a chi-square test within participants to determine whether the predicted proportion of responses from the model for each combination of distractor durations was statistically different from the observed ones (Fig. 6b). For most participants (14 out of 19), we found no significant difference between predicted and observed data. See Table 1 for a list of the fits for each participant.

**Table 1:** Chi-square test parameters by participant.

ID	χ^2^(df, N)	*p*
1	31.49 (11,30)	< .05
2	13.01 (11,30)	.293
3	7.54 (11,30)	.754
4	21.74 (11,30)	< .05
5	8.55 (11,30)	.664
6	11.52 (11,30)	.401
7	39.5 (11,30)	< .05
8	18.4 (11,30)	.073
9	9.62 (11,30)	.564
10	7.4 (11,30)	.766
12	14.85 (11,30)	.190
13	30.72 (11,30)	< .05
15	23.27 (11,30)	< .05
16	7.32 (11,30)	.773
17	7.33 (11,30)	.772
18	5.5 (11,30)	.905
19	3.33 (11,30)	.986
20	17.91 (11,30)	.084
21	14.57 (11,30)	.203

### Repulsion effect

**Figure 7:**
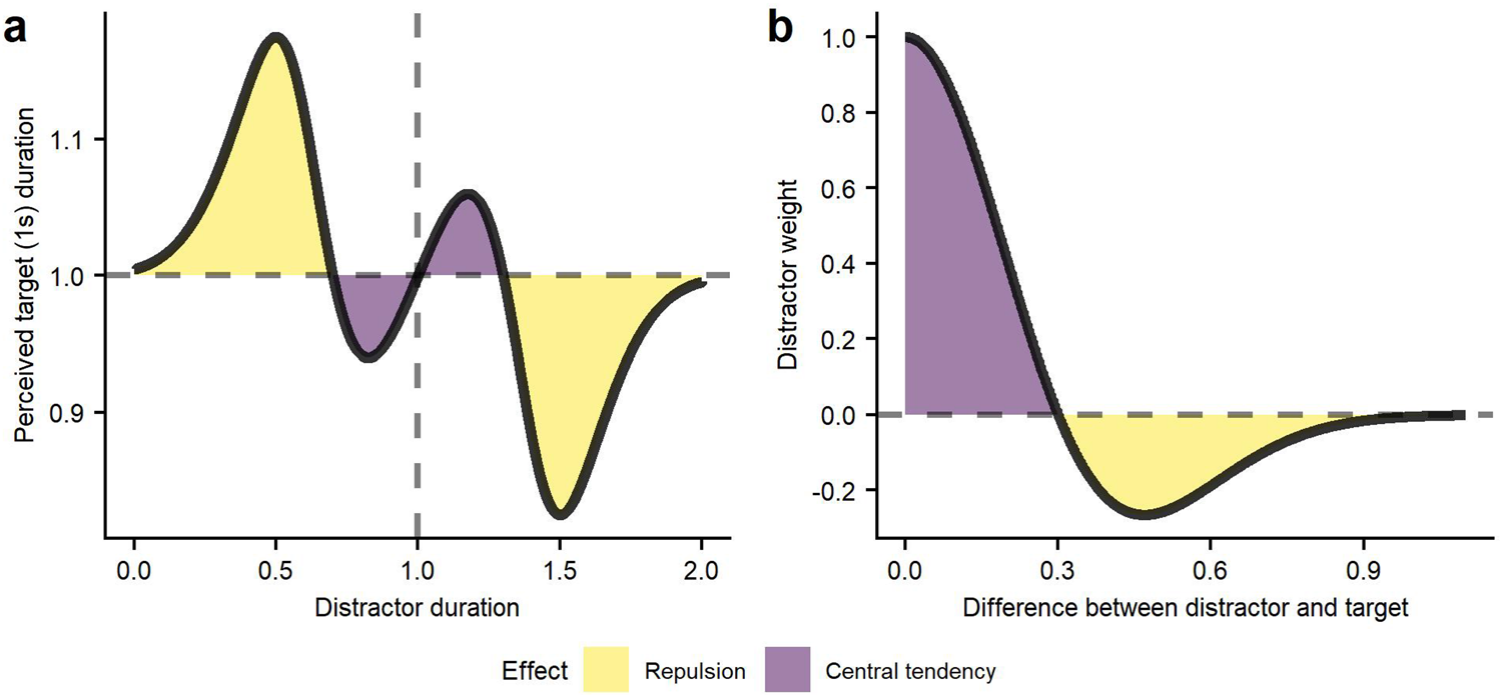
Example of the advanced model with a *k* of 0.3. (**a**) Perceived duration of a 1 s target as a function of the duration of simultaneous distractors. (**b**) Weight of the distractors as a function of their difference with the target duration using a ricker wavelet. Colours represents the direction of the effect from distractors on the perceived target duration.

Our model rooted in the duration channel strictly predicts a central tendency. However, in order to account for those participants that showed a repulsion tendency, it would be possible to postulate some inhibition between duration channels. We explored this idea and tested an extended version of the leaking function that uses a Ricker wavelet to compute the weight function:

7 $$w = \left( {1 - \left( {\frac{d - t}{k}} \right)^{2} } \right)\cdot2^{{ - \frac{{\left( {d - t} \right)^{2} }}{{k^{2} }}}} ; k > 0$$

where the weight (*w*) ranges from − 0.265 to 1 as a function of the difference between distractor (*d*) and target (*t*) durations. This is all modulated by the leaking factor (*k*), that can take any value greater than 0, and represents the difference in duration at which the effect would shift in direction (See Fig. 7).

This weight function would be approximated by a difference of two Gaussians (Mexican hat) and implements the effect of lateral inhibition^32,33^, which leads to central tendency for small differences between target and distractors and repulsion for larger values. The transition between central tendency and repulsion is defined by the *k* value in Eq. (7). Finally, distractors will have no effect at much greater values (See Fig. 7b).

**Figure 8:**
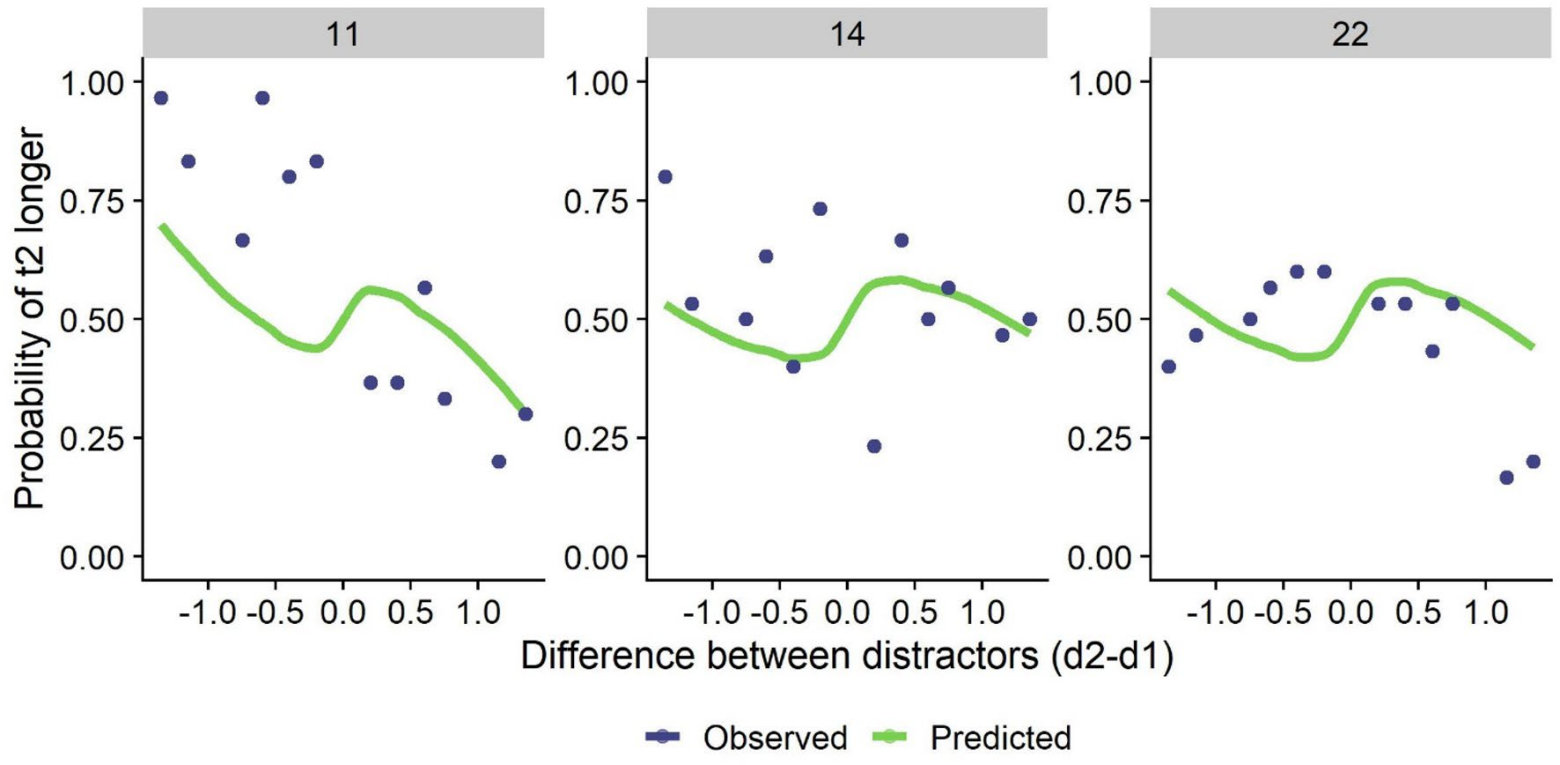
Fit between predicted and observed response probabilities as a function of the difference between distractors for participants with repulsion effect. Predicted probabilities were obtained with the alternative model of lateral inhibition.

We tested this alternative model following the same procedure as with the original model while keeping the same number of parameters. We did not, for example, modulate the differential effect of central average or repulsion. We found that the predicted responses obtained with the best fitting *k* estimate (using MLE) were not significantly different that the observed responses for two out of the three participants with repulsion (See Fig. 8 and Table 2). This suggests a good fit for some of the participants that were not able to be accounted for by the original leaking model.

**Table 2:** Chi-square test parameters by participant using the modified weight function.

ID	χ^2^(df, N)	*p*
11	31.85 (11,30)	< .05
14	17.99 (11,30)	.082
22	19.48 (11,30)	.053

### Sensitivity to noise

We also wanted to check whether the leaking factor is related to the capacity to filter out the effect of perceptual noise and preserve duration discriminability when the time intervals are composed of multiple elements rather than one. It could be relevant to know in which way the Channel Leaking model is related to how much information is integrated into the final estimation of the target interval. Hence, susceptibility to being affected by greater ranges of distractors could be related to the loss in discrimination capacity with more complex stimuli.

We used the ratio of variances from the Control and Ensemble conditions (σ^2^_Control_/σ^2^_Ensemble_) to represent the tolerance to perceptual noise. The variances can be obtained from the slopes of the psychometric curves in the two conditions. Values of this ratio closer to 1 indicate that duration judgements with multiple stimuli are as good as judgements with single stimuli, whereas values closer to 0 would indicate a substantial impairment in discriminability due to the increased number of stimuli in the scene.

**Figure 9:**
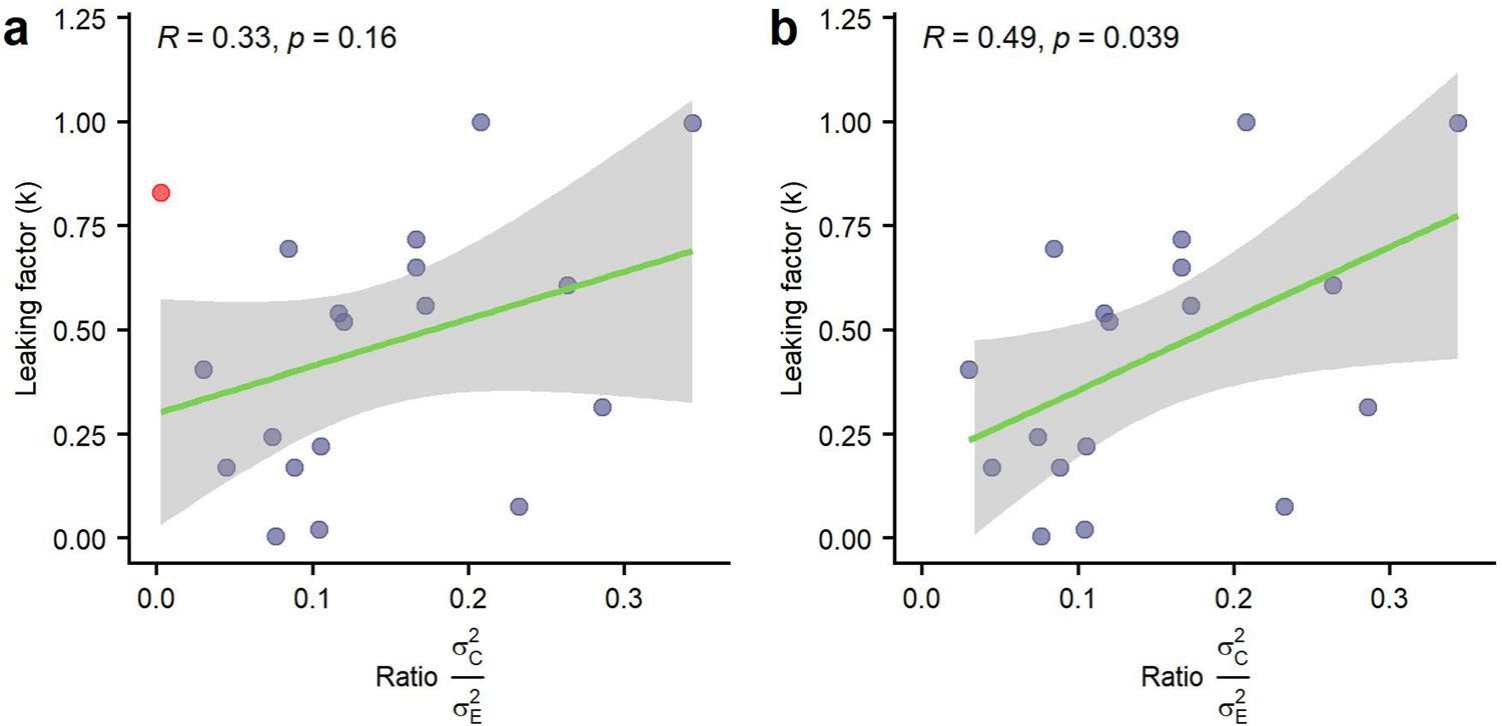
Correlation between leaking factor and ratio of variances. (**a**) Data from all participants with a central tendency. An outlier participant is highlighted in red. (**b**) Same correlation excluding the outlier participant.

Although we found a correlation between the ratio of variances and the leaking factor *r*(17) = 0.33, p = 0.165, it failed to reach significance. Nonetheless, excluding one participant that reported an extremely high sensitivity in the Control condition (final total sample of N = 18), we observed that the correlation became significant *r*(16) = 0.49, *p* = 0.039. See Fig. 9 for a comparison of the correlation with and without this participant.

Unexpectedly, this positive correlation is at odds with the expected relation between these variables. This trend shows that leaking factors tend to be greater when discriminability is less impaired by increasing the number of stimuli to be attended (larger variance ratios). This relation might suggest that the capacity of discriminating durations of more complex events, such as duration intervals constituted by multiple elements, is associated with a greater permeability of duration channels sensitive to different durations. When observers are more susceptible to integrating the duration of surrounding distractors, they are more capable of attending to the different stimuli composing an event. In other words, when participants are less capable of processing durations that require attention to multiple stimuli (which is indicated by a lower ratio of variances), they are less affected by multiple distractors (lower leaking factor) too.

## Discussion

The present study aimed to investigate how simultaneous temporal context could affect perceptual decisions about the duration of a task-relevant event. In a duration judgement task, our data show a clear effect of concurrent events (distractors) on the target duration. Additionally, a model is proposed to explain how this effect takes place. The information from the distractors is integrated into the perceptual decision process, and the model provides a good fit for the observed proportion of duration judgements.

We have reviewed different studies that have observed how recent or concomitant information could affect reproductions and duration judgments. Depending on the framework and paradigm, different types of effects have been reported. On the one hand, Bayesian studies predict that, under uncertainty, estimations tend to be biased towards an average of the contextual evidence^6–9^. Furthermore, other studies suggest that these interferences are revealed in simultaneous multiple timing tasks, usually following an averaging tendency^17–20^. Alternatively, adaptation studies often report the effect of accumulated contextual information as being a repulsive or contrastive one, in which the perceived durations of a target would be shifted away from previously presented durations^11–13^.

To address what would happen in a simultaneous duration paradigm, we used a perceptual decision task in which participants had to compare the duration of target stimuli overlapped in time with concurrent distractors. This task is motivated by the distractors affecting the perceptual appearance of duration, although some uncertainty may have been introduced since the two targets to be compared lasted the same. Therefore, any predominant bias should be related to the differences in duration of the simultaneous but irrelevant distractors that accompanied each target.

First, we compared the duration discrimination performance in the Ensemble and Control conditions. When participants had to discriminate durations of events made of multiple stimuli, performance was worse than when the same durations were bound to only one stimulus. Here we see the effect of what we attribute to be perceptual noise. Then, we wonder whether this perceptual noise could also have any effect when it is irrelevant to the task. Are we able to ignore this information? and, if not, in which way would it affect us?

We found that the perceptual noise from distractors in the Distractors condition, referred to as the temporal context, succeeded in producing an effect on the perceived duration of the target. Here, participants had to ignore the distractors and compare only the target, but their responses varied systematically with the durations of distractors.

The direction of this effect was mostly consistent with those studies reporting averaging tendencies with simultaneous information^17–19^. All participants but three showed a tendency to judge the duration of target stimuli as being more similar to the distractors that accompanied them. This outcome bodes well with the robustness of averaging that has been shown previously regardless of using a sequential or a simultaneous presentation paradigm^17,18,20^.

A small number of participants showed the opposite pattern, a repulsion type interference similar to those found in adaptation studies^11–13^, as they judged the target stimuli as lasting shorter in duration when distractors lasted longer and vice versa. This was unexpected and reveals that different tendencies can be observed using the same paradigm, pointing to some yet-to-be-known factor that interacts with our processing and judgements of duration.

To explain why these two outcomes can co-occur raises an important question for future investigations using an individual differences approach. One possibility is that, as reported from carryover effects studies, opposite directionalities of the effect can be determined by the cognitive nature of the interfering information, relating central tendency effects to decisional effects and repulsion or contrastive effects to perceptual factors^8,9^. Also, it has been shown that both sensory and decisional components contribute to biases measured by psychometric functions, especially when judgements involve spatial features^34^, so perhaps a combination of both factors could be taking place in this kind of task. Our data is inconclusive about which kind of bias (perceptual or decisional) is predominant in our task.

Regarding the small proportion of participants that show a repulsion type effect, it is questionable whether this is due to the same mechanisms acting in adaptation paradigms. Adaptation studies rely on the saturation of a sensorial representation of specific characteristics, while our stimulus presentation does not induce such sensorial saturation. In our case, the effect can appear with just one presentation, and no repetitions are required. Therefore, it may rely on mechanisms other than those intervening in adaptation effects.

These unexpected results highlight a limitation of the durations channels model. It is initially unable to predict repulsion effect, since it is formulated under the assumption of a central tendency, and calculated through a weighted average. To alleviate this issue, we put forward an alternative formulation of the weight function that allows for repulsion and central tendency effects to coexist due to lateral inhibition^32,33^. We were able to predict well the response pattern of some of those participants with repulsion effects. Although we explored a possibility to account for repulsion by modifying the weight function, we think that a full account of both central tendency and repulsion would need to be further addressed in future research and consider the contribution of perceptual and decisional components in each of these two opposite effects.

Assuming that each different stimulus activates respective channels that are selective to specific durations, we expected that, when presenting multiple stimuli, these channels could be activated by relevant and irrelevant stimuli. The question arises about how these parallel activations could later interact with each other. We demonstrate that the duration of distractors was somehow included in the computation of the perceived target duration. The information from these channels must be combined at some point in the computational process and modulated by the difference between target and distractor durations. The proposed model also allowed us to estimate the rate of this modulation as a decay parameter or leaking factor. The leaking factor determines the weight that information of distractors should have in the final estimation of the target.

We also highlight the considerable variability of leaking factor levels found between participants. Some observers are very good at ignoring distractors, others let some of these durations from distractors be integrated into the target estimations, and others even guide their decisions in our task almost entirely by the duration of distractors. Regarding the reason for such variability, it is possible that these differences in leaking factors could not happen just between observers but also vary within the same observer. For example, one reason for this sensibility to external information could be due to the uncertainty of the task. This points out one possible limitation of the study. Participants were asked to detect differences that did not actually exist but were induced by unattended external stimulation. Therefore, depending on how reliant participants were on surrounding stimuli, they could have been under different levels of uncertainty. This use of what could be considered a deceptive task could arguably be considered as unusual and frustrating for the participants, although from an informal debriefing, all participants reported that they believed they could discriminate between targets in most cases, and did not feel being deceived.

The core parameter of the model, *k*, might prove useful in future research since it could let us determine the range of durations one should or should not use when studying the effect of temporal contexts. Furthermore, the leaking function could be used even in paradigms different from the duration judgement task we conducted. It could be interesting to see how well it would fit other types of tasks such as duration reproduction, since we know that temporal context could present differential effects depending on the level of processing of the eliciting information^8,9^.

In addition to the need of an extended model to account for repulsion, another, probably more important aspect implied by the model as stated in Eq. (1) is whether or not Weber’s law holds. As commented before, the model does not predict a logarithmic perceptual scale between physical duration and perceived duration, but rather a linear one. This fact initially can compromise the model’s compatibility with Weber’s law, since when Weber’s law is integrated to build a perceptual scale, it results in a logarithmic one (i.e., Fechner’s law). However, the noise in such situation is assumed to be additive (i.e., independent of the physical duration). A linear perceptual scale can still be consistent with Weber’s law if we assume a multiplicative noise rather than additive noise^25^. To the best of our knowledge, we cannot know either the type of noise or the kind of perceptual scale (linear or logarithmic) from the time perception literature using discrimination tasks. However, it has been reported^35^ a nearly linear relation between perceived and physical duration with an exponent of 0.91 in Stevens’ Power law^36^ for duration estimation judgements. Although this would be close to a linear perceptual scale, further research would be needed to establish the compatibility of our model with Weber’s law.

Finally, we also wanted to check how each individual's capacity to discriminate between durations could prevent or enhance the magnitude of the temporal context effect. We expected the influence from distractors (measured by the leaking factor) to be smaller for participants with better discriminability. However, we found the opposite trend, not significant though, with good preservation of discriminability when events were composed of multiple stimuli associated with greater values of the leaking factor. This could allow a greater range of distractor stimuli to be integrated into a target's duration estimation.

The positive correlation between the capacity to preserve discriminability with more elements and the leaking factor raises important questions. Maybe the leaking of information between channels that we defined does not only describe the interference of distractors in the perceptual process but is also somehow an underlying feature of how humans integrate simultaneous duration information from multiple and more varied sources, both attended and unattended. We consider this a promising path for future research on multiple timing.

## Conclusion

We observed that duration judgments of a single event could be biased by the temporal context represented by the duration of simultaneous but irrelevant distractors. The outcome of this influence is, in most cases, a central tendency effect, where duration estimations are shifted towards a general average of all stimuli in the scene, regardless of their relevance to the task. In a few cases, this effect is reversed, and estimations are shifted away from the duration of distractors.

We provide a relatively simple model that predicts the influence of distractors by using a single parameter.

## Supplementary Information

Supplementary Figures.

## Data Availability

The original datasets are available at https://osf.io/yjcqr/.
