# Supplementary material for: Duration judgments are mediated by the similarity with the temporal context
Source: Sci Rep. 2022 Dec 30;12:22575. doi: 10.1038/s41598-022-27168-w (PMC9803681; doi:10.1038/s41598-022-27168-w)
Supplement: Supplementary file 1 — Supplementary Figures. [file 41598_2022_27168_MOESM1_ESM.pdf]

Supplementary Material

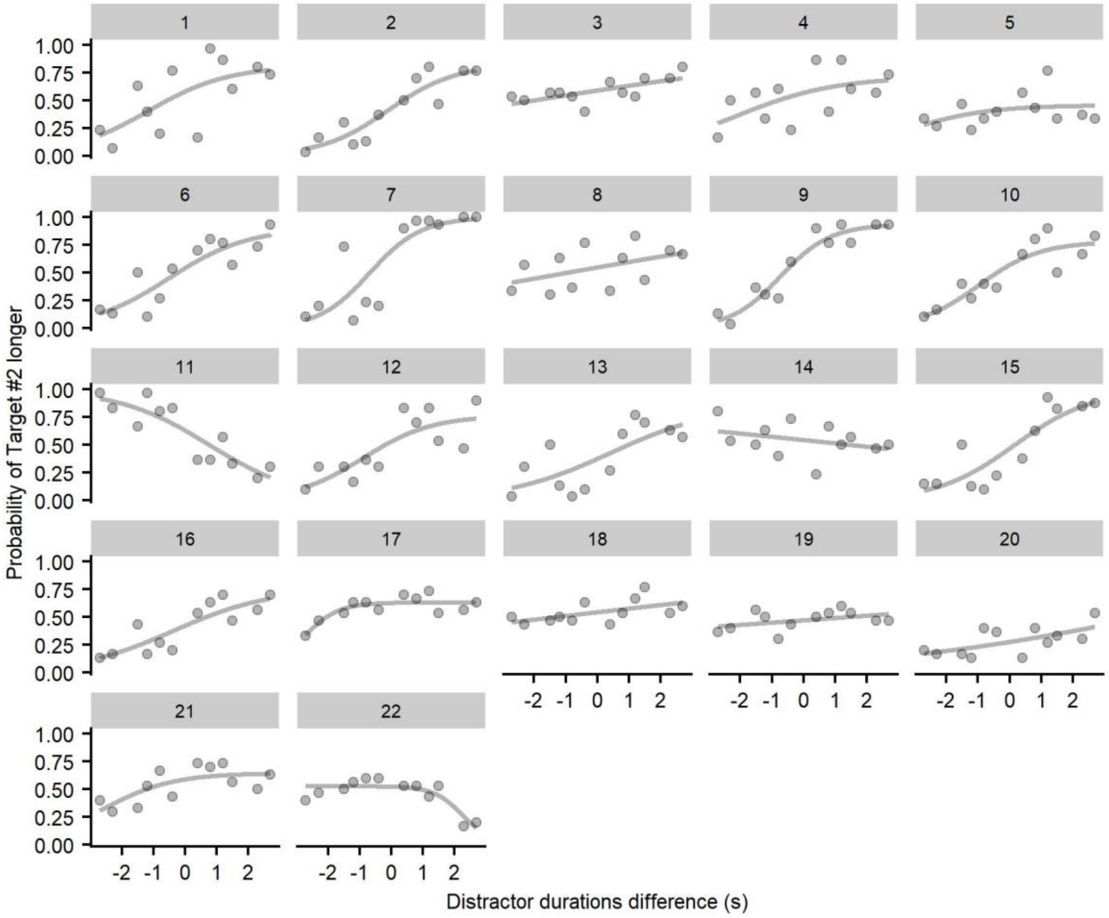

Supplementary Figure 1. Psychometric function of each participant. Probability of judging the target of the second sequence as longer as a function of the difference between distractor durations (second sequence – first sequence).

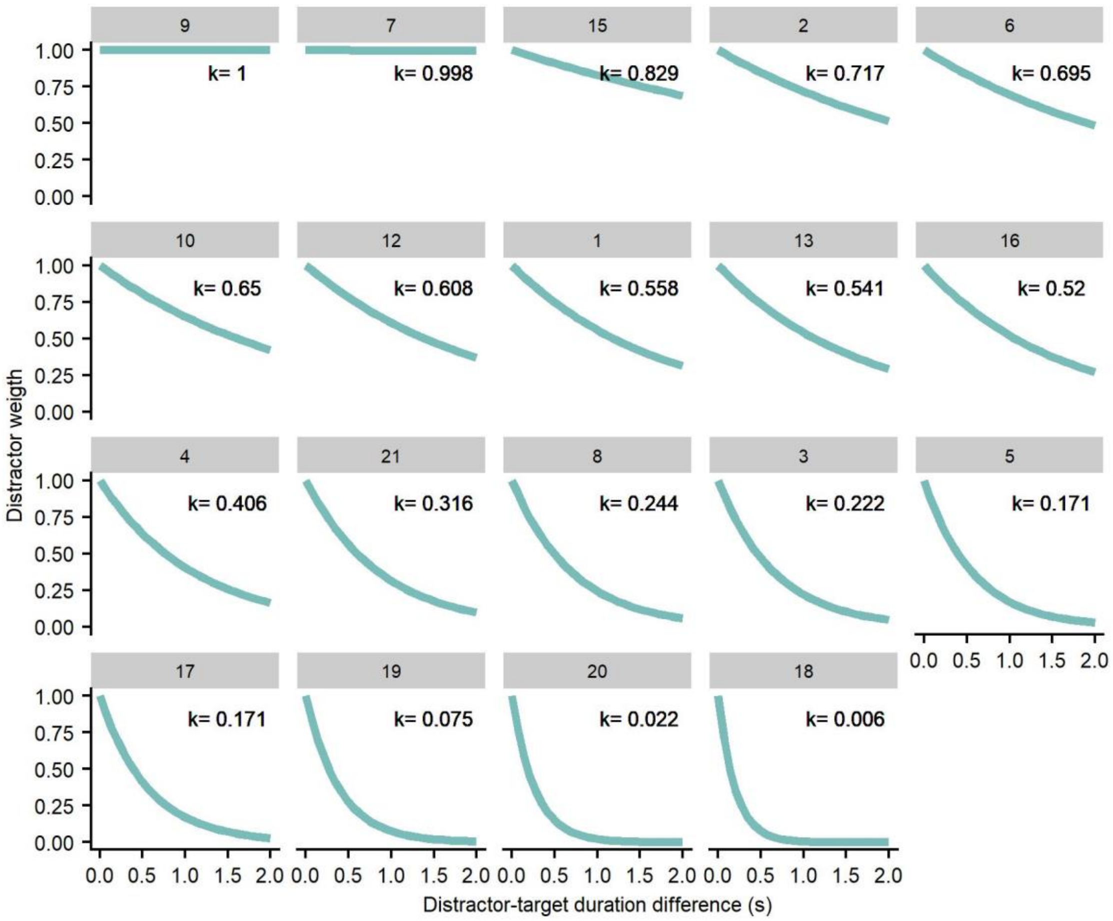

Supplementary Figure 2. Leaking function extracted for each participant.

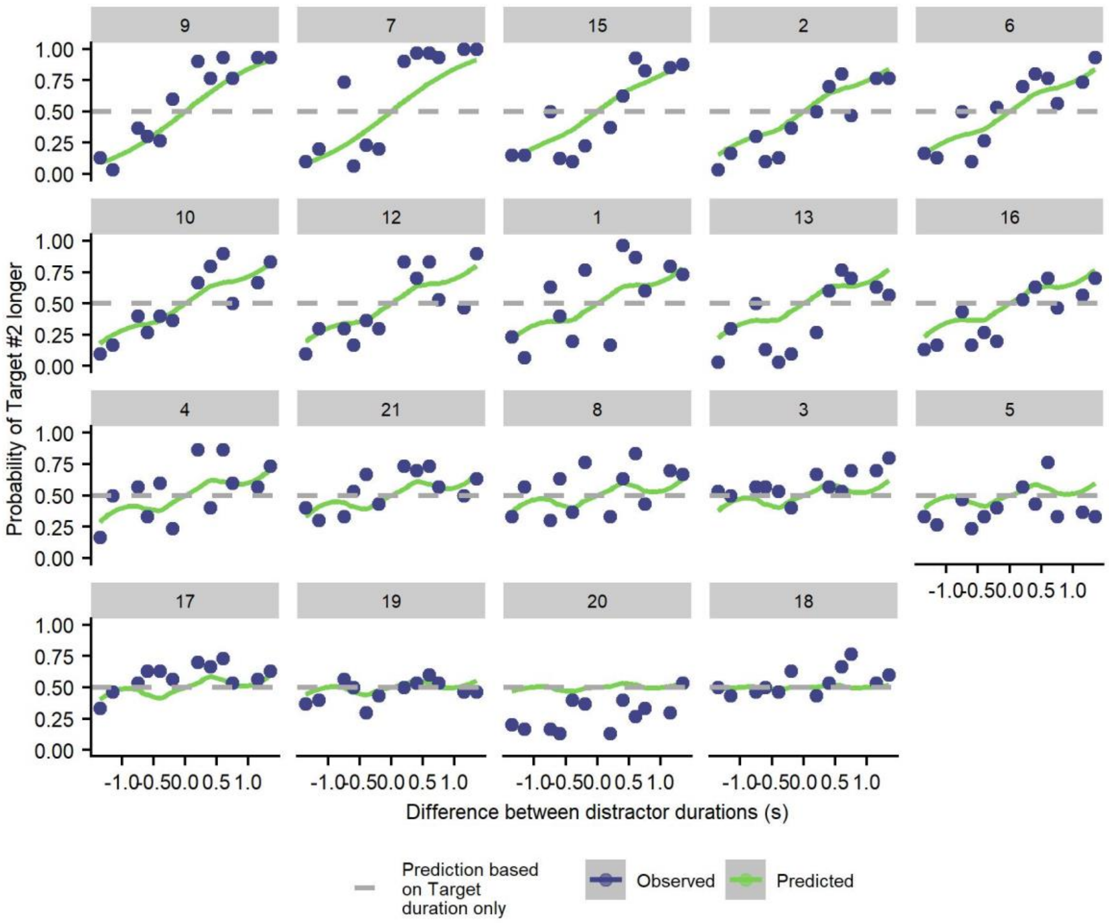

Supplementary Figure 3. Fit of predicted vs observed data using the weighted average function.

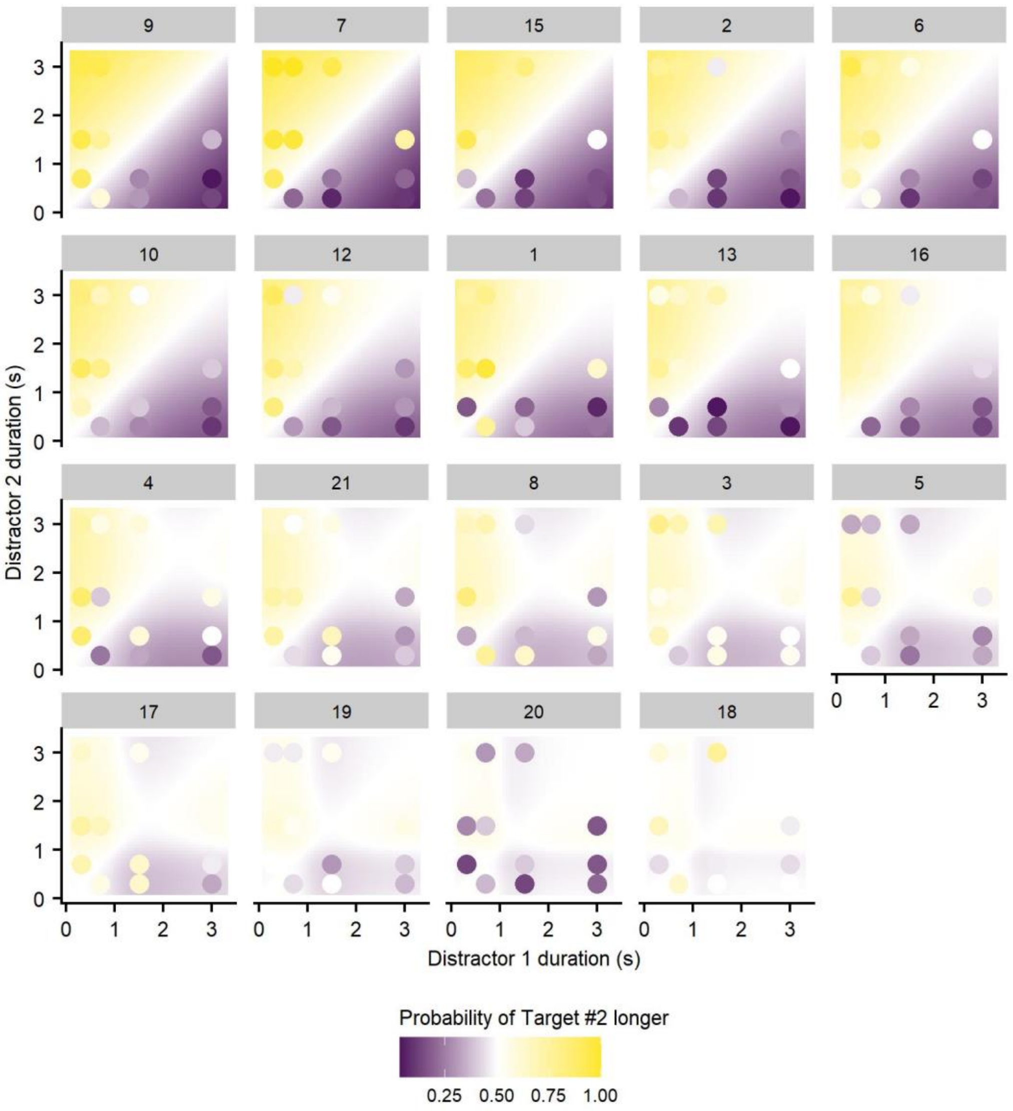

Supplementary Figure 4. Fit of the probability of response for each combination of distractors to the predicted probability obtained with the model.

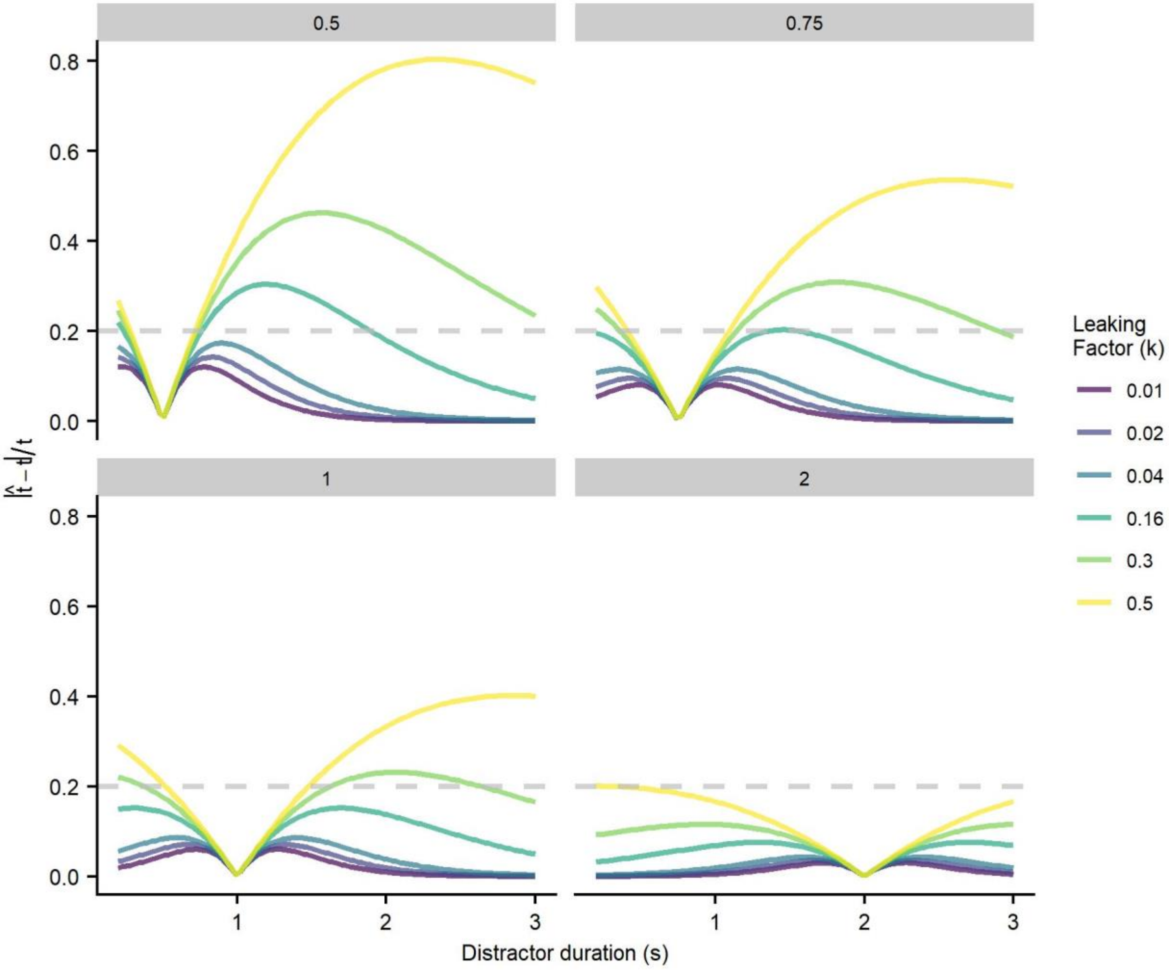

Supplementary Figure 5. Perceptual differences $(\hat{t} - t)$ divided by the reference time (t) after combining physical duration (t), in different panels, with distractor values (x-axis). Color codes different leaking factors. The grey dashed line corresponds to a Weber Fraction of 20%. Values above the grey line represent new perceived target durations that could be noticeably different from its physical duration after combining with the distractors. One can see the that the effect of the distractor on the perceived duration decreases as the distractors duration deviates from the reference time.
